# Anti-TNF Alpha Antibody Humira with pH-dependent Binding Characteristics: A constant-pH Molecular Dynamics, Gaussian Accelerated Molecular Dynamics, and In Vitro Study

**DOI:** 10.3390/biom11020334

**Published:** 2021-02-23

**Authors:** Shih-Ting Hong, Yu-Cheng Su, Yu-Jen Wang, Tian-Lu Cheng, Yeng-Tseng Wang

**Affiliations:** 1 Graduate Institute of Medicine, Kaohsiung Medical University, Kaohsiung 80708, Taiwan; gwu714@gmail.com; 2 Department of Biological Science and Technology, National Yang Ming Chiao Tung University, Hsin-Chu 300, Taiwan; ycsu-johnny@nctu.edu.tw; 3 Department of Mechanical and Electromechanical Engineering, National Sun Yat-sen University, Kaohsiung 80424, Taiwan; yjwang@mail.nsysu.edu.tw; 4 Drug Development and Value Creation Research Center, Kaohsiung Medical University, Kaohsiung 80708, Taiwan; 5 School of Post-Baccalaureate Medicine, College of Medicine, Kaohsiung Medical University, Kaohsiung 80708, Taiwan; 6 Department of Medical Research, Kaohsiung Medical University Hospital, Kaohsiung 80708, Taiwan

**Keywords:** constant-pH molecular dynamics, molecular simulations, antibody, anti-TNF alpha, Gaussian accelerated molecular dynamics

## Abstract

Humira is a monoclonal antibody that binds to TNF alpha, inactivates TNF alpha receptors, and inhibits inflammation. Neonatal Fc receptors can mediate the transcytosis of Humira–TNF alpha complex structures and process them toward degradation pathways, which reduces the therapeutic effect of Humira. Allowing the Humira–TNF alpha complex structures to dissociate to Humira and soluble TNF alpha in the early endosome to enable Humira recycling is crucial. We used the cytoplasmic pH (7.4), the early endosomal pH (6.0), and pK_a_ of histidine side chains (6.0–6.4) to mutate the residues of complementarity-determining regions with histidine. Our engineered Humira (W1-Humira) can bind to TNF alpha in plasma at neutral pH and dissociate from the TNF alpha in the endosome at acidic pH. We used the constant-pH molecular dynamics, Gaussian accelerated molecular dynamics, two-dimensional potential mean force profiles, and in vitro methods to investigate the characteristics of W1-Humira. Our results revealed that the proposed Humira can bind TNF alpha with pH-dependent affinity in vitro. The W1-Humira was weaker than wild-type Humira at neutral pH in vitro, and our prediction results were close to the in vitro results. Furthermore, our approach displayed a high accuracy in antibody pH-dependent binding characteristics prediction, which may facilitate antibody drug design. Advancements in computational methods and computing power may further aid in addressing the challenges in antibody drug design.

## 1. Introduction

Monoclonal antibodies are the most widely used treatment for autoimmune diseases, malignancies, and infectious diseases [1]. However, a monoclonal antibody is limited to binding to an antigen only once. Neonatal Fc receptor (FcRn) receptors can mediate the transcytosis of antibody–antigen complex structures and process them toward degradation pathways [2], reducing the therapeutic effect of antibodies [3]. Humira is a recombinant human IgG1 monoclonal antibody that binds to human TNF alpha and inhibits inflammation [4,5]. Humira is used to treat rheumatoid arthritis, psoriatic arthritis, ankylosing spondylitis, Crohn’s disease, plaque psoriasis, juvenile idiopathic arthritis, ulcerative colitis, and hidradenitis suppurativa [6]. Humira is useful in treating these autoimmune diseases but is extremely expensive, with an average cost of US$10,000–80,000 per year [7]. Humira’s steady-state volume of distribution, clearance, and serum half-life are 5.61 day^−1^, 0.22 l day^−1^, and 21 days, respectively [8]. Therefore, strategies to reduce clearance, prolong serum half-life, and increase the therapeutic effects are urgently needed. A new pH-switch strategy has been developed to reduce the clearance, prolong serum half-life, and increase the therapeutic effect of Humira [9]. Humira is limited because it can only bind to TNF alpha once. FcRn receptors mediate transcytosis of Humira-TNF alpha complex structures and process them toward degradation pathways. We aimed to use the cytoplasmic pH (7.4), early endosomal pH (6.0), and pK_a_ of histidine side chains (6.0–6.4) to mutate the residues of complementarity-determining regions (CDRs) with histidine residues. A study demonstrated that engineered Humira can bind to a TNF alpha in the plasma at neutral pH and dissociate from the TNF alpha in endosomes at an acidic pH [9]. The engineered Humira antibodies [9] lost 90–99% of their TNF alpha–binding abilities at pH 7.4, and these antibodies did not obviously dissociate from the TNF alpha in the endosome under an acidic pH (association rate constant: 0.67–1.93 × 10^6^ M^−1^ S^−1^ with pH at 7.4; disassociation rate constant: 4.8–11,000 × 10^−5^ S^−1^ with pH at 6.0). The key CDR positions replaced with histidine residues were detected at random using combinatorial histidine scanning libraries and phage display [10,11]. Antibody CDR3 loops (light or heavy complementarity-determining region 3) make dominant contributions to the antigen-binding affinity [12,13]. Studies have indicated that antibodies can have pH-dependent binding affinities after the key CDR3s positions are replaced with histidine residues [11,14,15]. However, no efficient and regulated method has been developed to equip Humira with pH-dependent TNF alpha-binding affinities. For above reasons, a reliable modeling strategy of antibodies with pH-dependent binding is urgent. 

For larger biomolecular systems, most conventional molecular dynamics (cMD) simulations aim to sample statistical mechanical ensembles using fixed-valence force-field models [16,17]. Biomolecular systems are regulated by carefully buffered solutions and a complex interplay between multiple protonation states, which is affected by enzyme sensitivity to pH conditions [18,19,20]. The number of states relevant to cMD simulation is relatively small. Therefore, cMD simulation can be studied through brute force enumeration. However, the cMD approach quickly becomes untenable for larger systems or even simple solutions with modest concentrations. 

A tight coupling between protonation equilibria and conformation exchanges is observed in antibodies, and the importance of solvent pH in MD simulations has been recognized. [21,22] In general, the solvent pH in MD has been limited to setting a constant protonation state for each titratable group in a biomolecule system. This approach has many drawbacks. First, assigning protonation states requires knowledge of pKa values for the protein’s titratable groups. Second, if any of these pKa values are near the solvent pH, there may be no single protonation state that adequately represents the ensemble of protonation states appropriate at that pH. Finally, because the assumed protonation states are constant, this approach decouples the dynamic dependence of pKa and protonation state on conformation. Constant pH molecular dynamics (CpHMD) approaches can overcome the issue and also be used for large biomolecular systems, which can achieve the desired pH condition [23,24,25,26,27]. CpHMD simulations involving carboxyl and histidine residues have been in agreement with experiments in the turkey ovomucoid third domain and ribonuclease A [25].

The application of an all-atom molecular dynamics simulation to study conformational ensembles obtained from a single, long-time-scale cMD simulation remains limited; this limitation may be caused by energy barriers between various intermediate states. Therefore, an enhanced sampling technique was required for this task. Enhanced sampling techniques have been successfully applied to evaluate binding mechanisms and structural dynamics [28], including the metadynamics method [29], adaptive biasing force method [30] with coarse-grained conformational sampling, cMD [31], accelerated molecular dynamics (aMD), and Gaussian accelerated molecular dynamics (GaMD) [32]. These enhanced sampling studies provide valuable insights into binding mechanisms and structural dynamics. The disadvantage of enhanced sampling techniques is the requirement for predefined parameters (i.e., root-mean-square distance and protein structures). However, aMD (or GaMD) can be used as an enhanced sampling method. In the aMD method, a boost potential is added to the potential energy surface; the energy barriers are thus effectively reduced, accelerating transitions between low-energy states [32,33,34]. The aMD method has also been successfully applied to biological system simulations, and aMD simulations performed on the time scale of hundreds of nanoseconds can approach cMD simulations performed on the millisecond timescale [35,36,37,38]. A drawback of the aMD method is the large energetic noise occurring during reweighting [39]. In aMD simulations, the applied boost potential is typically in the order of tens to hundreds of kilocalories per mole (kcal/mol), which is much higher than that of enhanced sampling methods that use protein structures or reaction coordinates. Accurately reweighting aMD simulations is difficult, particularly for large protein molecules [40]. Miao et al. presented a solution (i.e., GaMD) for improving the aMD method. In the GaMD method, the boost potential follows a near-Gaussian distribution, and cumulant expansion to the second-order improves the reweighting of aMD simulations [41]. The reweighted free energy profiles of GaMD accord with those of the long-time-scale cMD simulations [42]. In this study, we employed CpHMDs, GaMD, two-dimensional (2D) potential mean force profiles, and in vitro methods.

## 2. Materials and Methods

**Figure 1:**
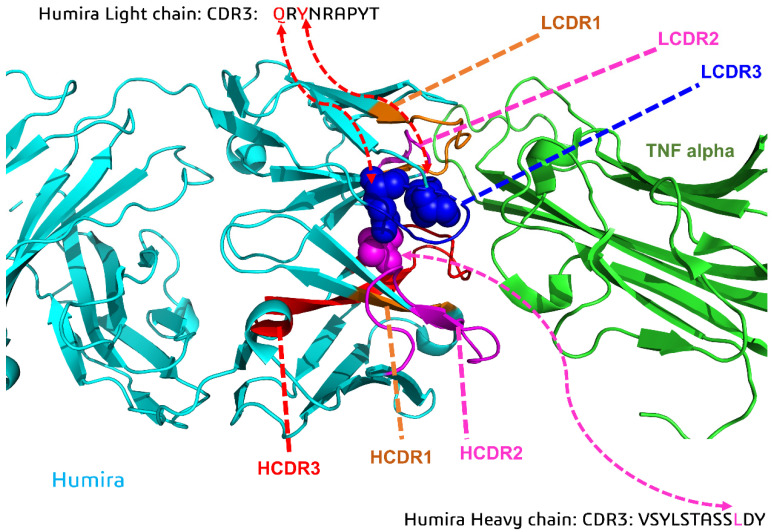
Overview of the key CDR (light and heavy complementarity-determining region 3, HCDR3, and LCDR3) positions replaced with histidine residues (W1-Humira).

Our strategy comprised the following steps:

Step 1: Identify the key residues of CDRs (PDB ID: 4NYL, adalimumab), which were mutated with histidine residues. If the distance between the center of mass of the two residues from the different CDRs was less than 10 Å, the two residues were defined as the key residues (Figure 1).

Step 2. Build an MD simulation model (leaprc.constph) on tleap.

Step 3. Perform energy minimizations, NVT (1 ns), and NPT (1 ns) equilibration using pmemd.cuda.

Step 4. Perform a 20-ns GaMD/CpHMD equilibration using pmemd.cuda.

Step 5. Perform four 300-ns GaMD/CpHMD production simulations using pmemd.cuda.

Step 6. Perform analysis of the trajectory using CPPTRAJ to obtain the 2D free energy with the PyReweighting toolkit.

Step 7. Compare the complex structures with lower potential of mean force (PMF) values with the original antibody (PDB ID: 4NYL)

Step 8. Perform four 200-ns CpHMD production simulations using pmemd.cuda.

### 2.1. System Setup

**Figure 2:**
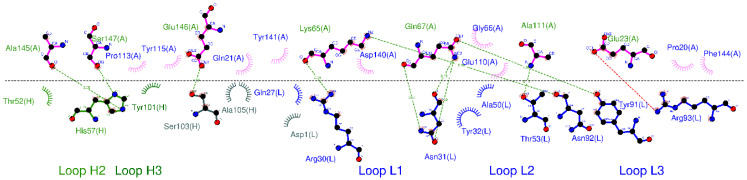
Binding mode analysis of Humira–TNF alpha (PDB ID: 3WD5; H: heavy chain; L: light chain; A: TNF alpha).

For the initial antibody Humira model (PDB ID: 4NYL, adalimumab), the key CDR (light and heavy complementarity-determining region 3, HCDR3, and LCDR3) positions were replaced with histidine residues (Figure 1, W1-Humira). The structures were then generated (size: approximately 9.32 × 9.32 × 9.32 nm^3^) and inserted into TIP3P solvent molecules. These initial complexes were then simulated using the software package AMBER 18 with the all-hydrogen amino acid AMBER constant pH force field (leaprc.constph). For the simulation, the pH conditions were 6.0 and 7.4. All CpHMD simulations were performed in the isothermal–isobaric (NPT) ensembles with a simulation temperature of 310 K, unless otherwise stated, using the Verlet integrator with an integration time step of 0.002 ps and SHAKE constraints [43] for all covalent bonds involving hydrogen atoms. In the electrostatic interactions, atom-based truncation was performed using the PME [44] method, and the switch van der Waals function was used with a 2.00-nm cutoff for atom-pair lists. These complex structures were minimized for 100,000 conjugate gradient steps and then subjected to 1-ns NVT and 1-ns NPT MD simulations. The final structures from these simulations were used in 20-ns GaMD/CpHMD equilibration and 300-ns GaMD/CpHMD production simulation calculations [41]. The simulation trajectories were recorded every 0.2 ps for analysis. Snapshots of all four 300-ns GaMD/CpHMD production simulations were used to calculate the backbone root-mean-square deviations (RMSDs) of the CDRs and the distances between the centers of the HCDR3 and LCDR3 using CPPTRAJ [45]. The PyReweighting toolkit was employed to reweight the GaMD/CpHMD simulations and calculate the PMF profiles of each antibody system [46]. These CDRs were selected as the 2D PMF reaction coordinates because of the binding modes analysis (Figure 2). The backbone reference RMSDs (BRRMSD) of the CDRs (HCDR2-3 and LCDR1–3) and the distances (D3) between the centers of the HCDR3 and LCDR3 were used as reaction coordinates. Complex structures with relatively low PMF values were selected for conformation analysis and subjected to 200-ns CpHMD equilibrations at pH 6.0 and 7.4.

### 2.2. GaMD

GaMD is an enhanced conformational sampling method for biomolecules that adds a harmonic boost potential to smooth the potential energy surface [41]. When the system potential (V) is lower than the referenced energy (E), a harmonic boost potential (ΔV) is added, as follows:

(1) $$\Delta V=\frac{1}{2}K\;{\left(E-V\right)}^{2},\;\mathrm{if}\;V<E,$$

where K is a harmonic force constant. The modified system potential (V*) can be described as follows:

(2) $$V^{*}=V+\frac{1}{2}K\;{\left(E-V\right)}^{2},\;\mathrm{if}\;V<E,$$

where if V > E, ΔV (harmonic boost potential) = 0. By smoothing the potential energy surface to overcome intermediate energy barriers, the boost potential satisfies the following step. For two potential energy values V1 and V2, assume that V1 < V2 and that the biased V1* < V2*. By replacing V* with Equation (2), the relationship is expressed as follows:

(3) $$E<\frac{1}{2}\left(V1+V2\right)+\frac{1}{K}.$$

Step (1): If V1 < V2, the potential difference on the smoothed energy surface should be smaller than that on the original energy surface. By replacing V* with Equation (2), the relationship is expressed as follows:

(4) $$E>\frac{1}{2}\left(V1+V2\right).$$

Step (2): By combining Equations (3) and (4) and including the relationship Vmin ≤ V1 < V2 ≤ Vmax, the following equation can be derived:

(5) $$\mathrm{Vmax}\leq E\leq \mathrm{Vmin}+\frac{1}{K},$$

where Vmin and Vmax are the minimum and maximum potential energy.

Step (3): From Equation (5), the following equation is obtained:

(6) $$\frac{1}{K}\leq \frac{1}{\mathrm{Vmax}-\mathrm{Vmin}},$$

where the K constant is defined as follows:

(7) $$K=K0\left(\frac{1}{\mathrm{Vmax}-\mathrm{Vmin}}\right),\;0<K0\leq 1,$$

where K0 is the magnitude of the applied boost potential.

Step (4): The standard deviation (SD) of ΔV must be sufficiently small to ensure accurate reweighting [46]:

(8) $$\sigma_{\Delta V}=\sqrt{{\left(\frac{\partial \Delta V}{\partial V}|V=\mathrm{Vave}\right)}^{2}\sigma_{V}{}^{2}}=K\;\left(E-\mathrm{Vave}\right)\sigma_{V}\leq \sigma_{0},$$

where Vave and σV are the average and SD of the potential energies, respectively, and σΔV is the SD of ΔV with σ0 as the user-specified upper limit for accurate reweighting of potential energies. In our simulations, the SDs of the total potential and dihedral potential boosts were 10 kcal/mol.

Step (5): To extend step (2) if E = Vmax, Equation (5) can be used to obtain

(9) $$K0\leq \frac{\sigma 0}{\sigma V}\frac{\mathrm{Vmax}-\mathrm{Vmin}}{\mathrm{Vmax}-\mathrm{Vave}}.$$

According to Equations (6) and (7), K0 can be defined as follows:

(10) $$K0=\min\left\{1.0,\frac{\sigma 0}{\sigma V}\frac{\mathrm{Vmax}-\mathrm{Vmin}}{\mathrm{Vmax}-\mathrm{Vave}}\right\}.$$

Step (6): To extend step (2) if E = Vmin + 1/k, Equation (8) can be used to obtain

(11) $$K0\geq \left(1-\frac{\sigma 0}{\sigma V}\right)\frac{\mathrm{Vmax}-\mathrm{Vmin}}{\mathrm{Vmax}-\mathrm{Vave}}.$$

Step (7): GaMD provides the total, dihedral, and dual potential boosts to accelerate molecular simulations. The boost potential (ΔV) is given as follows:

(12) $$\Delta V=\frac{1}{2}K0\frac{1}{V_{\max}-V_{\min}}{\left(E-V\right)}^{2},\;\mathrm{if}\;V<E,$$

where K0 is the magnitude of the applied boost potential, and Vmin and Vmax are the minimum and maximum potential energy of the system, respectively. Initially, K0 = 1.0, and Vmax and Vmin were obtained through cMD simulations. The distribution and anharmonicity of the GaMD method were applied to the alanine dipeptide, chignolin, and lysozyme simulations to characterize the extent to which ΔV follows a Gaussian distribution [41].

### 2.3. CpHMD

The CpHMD [47] method employs an extended Hamiltonian to predict the coordinates of the fictitious λ particles representing the protonation and deprotonation on the titratable site.

(13) $$\tilde{\lambda }=\left\{\begin{array}{c} 1\;\left(deprotonated\right),\;if\;\lambda >\;{\mathrm{cut}}_{1}\;\\ 0\;\left(protonated\right),\;if\;\lambda <\;{\mathrm{cut}}_{0} \end{array}\right.$$

where cut_1_ and cut_0_ are 0.8 and 0.2, respectively. The Hamiltonian, HB_j_ ⇌ B_j_, becomes

(14) $$H\left(\left\{r_{b}\right\},\;\left\{\lambda_{j}\right\}\right)=\sum_{b}^{N_{atom}}\frac{1}{2}m_{b}{\dot{r}}_{b}{}^{2}+\;U^{bond}\left(\left\{r_{b}\right\}\right)+U^{unbond}\left(\left\{r_{b}\right\},\;\left\{\lambda_{j}\right\}\right)+\sum_{j}^{N_{titration}}\frac{1}{2}m_{j}{\dot{\lambda }}_{j}{}^{2}+U\times \left(\left\{\lambda_{j}\right\}\right),$$

where the kinetic energies are displayed in the first and fourth terms, the λ independent bonded energies are displayed in the second term, and the λ dependent electrostatic and van der Waals energies are displayed in the third term. The atomic partial charges on the titratable site are defined as follows:

(15) $$q_{b,j}=\lambda_{j}\times q_{b,j}^{deprotonated}+\left(1-\lambda_{j}\right)\times q_{b,j}^{protonated}.$$

The electrostatic and van der Waals energies involving titratable hydrogens are linearly scaled by λ. The fourth term of Equation (13) contains three biasing potential energies.

(16) $$U\times \left(\left\{\lambda_{j}\right\}\right)=\sum_{j}^{N_{titration}}\left[-U^{mod}\left(\lambda_{j}\right)+U^{pH}\left(\lambda_{j}\right)+U^{barr}\left(\lambda_{j}\right)\right].$$

*U^mod^* is the PMF function for titrating a model compound in solution. The *U^mod^* term is as follows:

(17) $$U^{mod}\left(\lambda_{j}\right)=\;A_{j}{\left(\lambda_{j}-B_{j}\right)}^{2},$$

where the *A_j_* and *B_j_* parameters can be defined using a fitting procedure [47]. The *U*^pH^ term is used to calculate the deprotonation free energy change in the solution pH.

(18) $$U^{pH}\left(\lambda_{j}\right)=\;\ln\left(10\right)\times k_{B}T\times \left(pK_{a}^{model}-pH\right)\times \lambda_{j},$$

where $k_{B}\;$is the Boltzmann constant, *T* is the temperature, and $pK_{a}^{model}\;$is the model *pK_a_*, which can be identified from experiments. The final term of Equation (15) reduces the probability of λ in the unphysical intermediate state.

(19) $$U^{barr}\left(\lambda_{j}\right)=\;4\beta {\left(\lambda_{j}-0.5\right)}^{2},$$

where *β* is the height of the energy barrier.

### 2.4. Construction, Expression, and Antigen-Binding Ability of pH-Dependent Humira

The light chain and heavy chain sequences of W1-pH-dependent Humira were gene synthesized and then subcloned with IRES into the expression vector. Expression of W1 pH-dependent Humira was performed using Lipp2000 transfection reagent. TNFα was coated onto 96-well plates and blocked with 5% skim milk to investigate the antigen-binding of W1-pH-dependent Humira at pH 7.4 and 6. W1-pH-dependent Humira was added onto the plates at concentrations of 4.9–1,200 ng/mL at room temperature (RT) and then centrifuged at 50 rpm for 1 h. The Humira was then incubated in pH 7.4 (25 µM NaH_2_PO_4_ + 76 µM Na_2_HPO_4_) or pH 6 wash buffer (82 µM sodium citrate + 18 µM citrate acid) at RT and then centrifuged at 50 rpm for 30 min. The plates were then stained with HRP-goat antihuman IgG Fcγ antibody at RT, centrifuged at 50 rpm for 1 h, washed, and then color developed with ABTS containing 30% H_2_O_2_ (Sigma–Aldrich). The binding ability was quantified using absorbance detection at 405 nm.

## 3. Results

### 3.1. Prediction of Possible W1-Humira Conformations through GaMD/CpHMD Simulations

**Figure 3:**
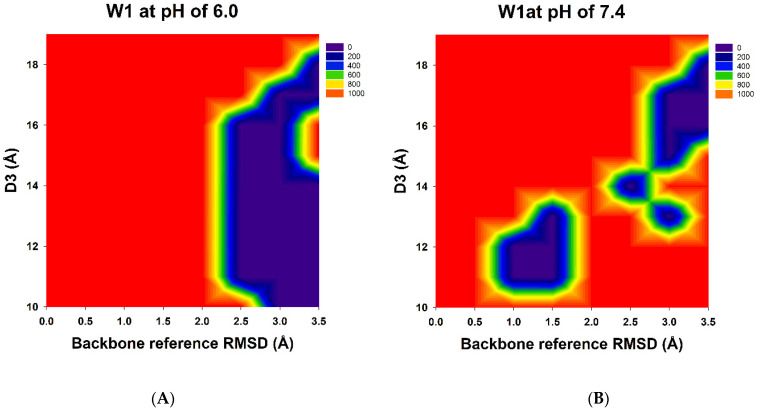
The 2D PMF profiles of the backbone reference root-mean-square deviations (BRRMSD) of the CDRs (HCDR2-3 and LCDR1–3) and the distances (D3) between the centers of the HCDR3 and LCDR3 were used as reaction coordinates: (**A**) W1-Humira at pH 6.0 and (**B**) W1-Humira at pH 7.4.

GaMD simulations can be used to refine protein conformations effectively. The 2D PyReweighting toolkit was applied to reweight the GaMD simulations. The 2D PMF profiles are illustrated in Figure 3. The 2D PMF calculations revealed that the protein structures exhibited higher PMF values and that the structures were unstable. The 2D PMF calculations also revealed possible free Humira antibody conformations with lower PMF values (local minima) at pH 6.0 and 7.4. We used the 2D PMF profile information to identify complex structures with lower PMF values (less than 50.0 kcal/mol) because these structures may be possible and reasonable. For W1-Humira at pH 6.0, the lower PMFs were located at over 2.0 Å (the backbone reference RMSD: BRRMSD) and the D3 of 10–18 Å (Table 1). We observed 20 conformation states with the lower PMF values, and the BRRMSDs of the 20 conformation states were highly different from the BRRMSD of wild-type Humira, in which the BRRMSDs were above 2.0 Å. Our predictions indicated that these conformation states could not bind the TNF alpha proteins at pH 6.0. At pH 7.4, the lower PMFs were located in the two major areas (Table 2). The first area was at the BRRMSD of 1.0–1.5 Å and D3 of 11–13 Å. Five conformation states with lower PMF values were identified, and the BRRMSDs of the five conformation states were highly similar to those of the BRRMSD of wild-type Humira, in which the BRRMSDs were at less than 1.5 Å. Therefore, our prediction indicated that these conformation states might bind the TNF alpha proteins at pH 7.4. The second area was BRRMSD of 2.5–3.5 Å and D3 of 13–19 Å. We identified 12 conformation states with lower PMF values. These conformation states with lower PMF values were selected for comparison with our CpHMD simulations. Eight conformation states displayed lower PMF values, and the BRRMSDs of the eight conformation states were highly different from the BRRMSD of the wild-type Humira, in which the BRRMSDs were at more than 2.5 Å. Therefore, our predictions indicated that these conformation states could not bind the TNF alpha proteins at pH 7.4. In summary, the 5/13 of W1-Humira may have the potential to bind TNF proteins at pH 7.4, and none of the W1-Humira appear to have the potential to bind TNF proteins at pH 6.0. Therefore, GaMD/CpHMD simulations generated significantly refined antibody conformations and provided possible conformation state information for predicting the binding characters at various pH values.

### 3.2. CpHMD Simulations and Binding Modes Analysis of W1-Humira and Wild-Type Humira at pH 6.0 and 7.4

**Figure 4:**
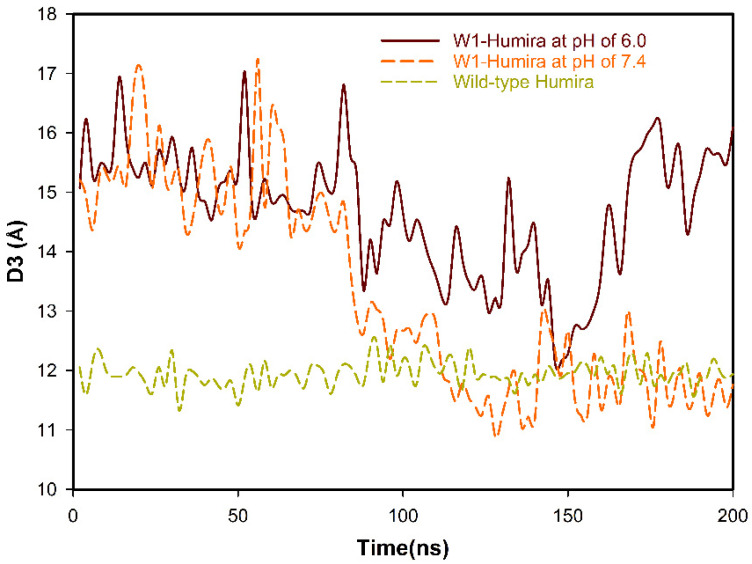
D3 distance profiles of the 200-ns CpHMD MD simulations for W1-Humira and wild-type Humira at pH 6.0 and 7.4.

**Figure 5:**
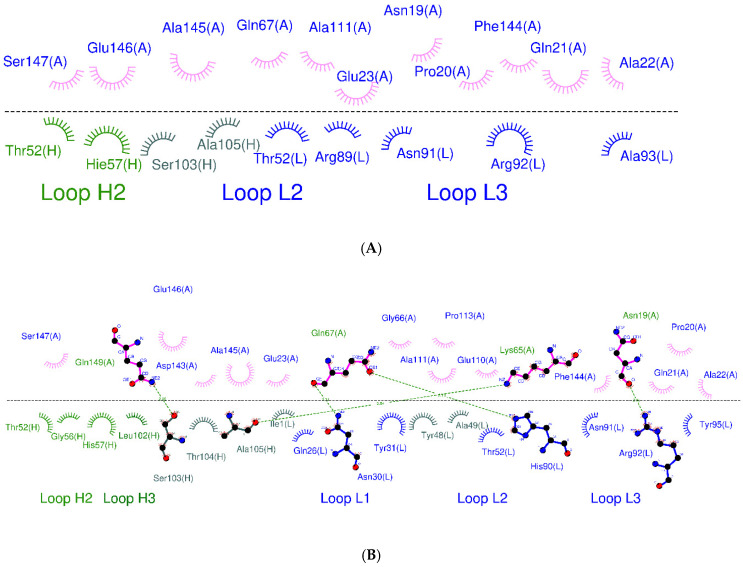
Binding mode analysis of W1-Humira–TNF alpha. (**A**) W1-Humira at pH 6.0 and (**B**) W1-Humira at pH 7.4. (H: heavy chain; L: light chain; A: TNF alpha).

CpHMD simulations can effectively predict protein conformations at various pH values. The wild-type Humira and W1-Humira were used in 200-ns CpHMD simulations to assess the GaMD/CpHMD simulation. The distances (D3) between the centers of the HCDR3 and LCDR3 were applied to study the CpHMD simulations, and the D3 distance profiles are illustrated in Figure 4. For W1-Humira in pH 6.0 CpHMD simulations, the D3 distance of 15 Å differed considerably from the D3 distance of the wild-type Humira. The D3 distance of W1-Humira in pH 7.4 CpHMD simulations was highly similar to the D3 distance of the wild-type Humira after the 100-ns CpHMD simulations, at 12 Å. The D3 distance of wild-type Humira in CpHMD simulation remained at 12 Å. Therefore, CpHMD simulations revealed the antibody conformation changes under different pH values. An attempt was made to predict the binding modes for W1-Humira with TNF alpha and investigate whether the CDR loops of W1-Humira bounded with TNF alpha. Binding modes were identified by aligning with the complex crystal structure (PDB ID: 3DW5), and the binding modes are illustrated in Figure 5 and Table 3.

### 3.3. In Vitro W1-Humira and Wild-Type Humira Binding Ability Testing at pH 6.0 and 7.4

**Figure 6:**
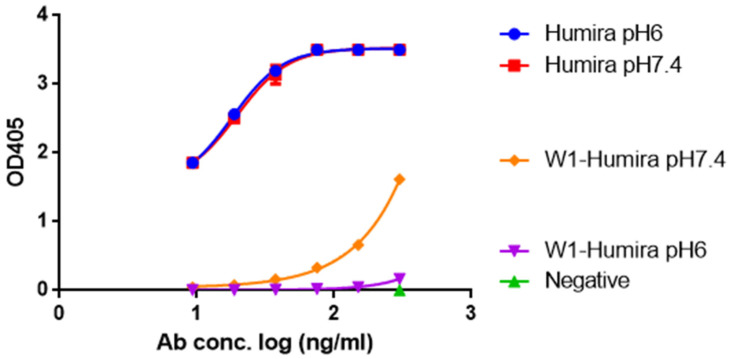
The TNF alpha-binding ability of W1-Humira and wild-type Humira at pH 6.0 and 7.4.

To determine whether W1 and wild-type Humira are pH-dependent or independent antibodies that recognize TNF alpha proteins, we examined the OD405 absorbance generated from the human TNF alpha ELISA kit with precoated plates, incubated proteins with pH 7.4 wash buffer (25 µM NaH_2_PO_4_ + 76 µM Na_2_HPO_4_) and pH 6 wash buffer (82 µM sodium citrate + 18 µM citrate acid), respectively. Our results indicated that W1-Humira is a pH-dependent antibody, whereas wild-type Humira is not (Figure 6). The OD405 absorbance at pH 6.0 was also extremely close to zero, indicating that the TNF alpha-binding function of W1-Humira was turned off. The W1-Humira is 0.5-fold weaker than wild-type Humira at pH 7.4 in vitro.

## 4. Discussion

The traditional strategy used to generate pH-dependent binding antibodies is to replace the key CDR positions of the antibodies with histidine residues selected at random with combinatorial histidine scanning libraries and phage display [48,49]. Researchers require several months or years to achieve their goals, and the binding affinities of their engineered antibodies are often weaker than the wild-type antibodies. Computer simulations can speed up antibody drug design [50]. Although molecular simulation techniques are applied in the design of pH-dependent binding antibodies, these methods cannot provide accurate predictions of antibody binding characteristics at various pH values, and these methods can only be used to perform simulations with X-ray crystals or to predict the complex structures of antibody Fab fragments and Fab–antigen complexes [51,52]. Therefore, antibody drug design remains a considerable challenge.

CpHMD approaches can be used for large biomolecular systems and can achieve the desired pH condition [23,24,25,26,27]. GaMD results accord with those of long-time-scale normal MD simulations [42]. Therefore, we combined the CpHMD and GaMD methods to simulate our W-1 Humira antibody at pH 6.0 and 7.4. The 2D PMF profiles may indicate the pH-dependent binding characteristics of W1-Humira and wild-type Humira at pH 6.0 and 7.4. We used the information from the 2D PMF profiles (Figure 3 and Table 1 and Table 2) to determine the antibody structures with low PMF values (less than 50.0 kcal/mol) because these may be possible and reasonable structures. The results of the GaMD/CpHMD simulations of W1-Humira indicated that these conformation states could not bind the TNF alpha proteins at pH 7.4. Therefore the 5/13 of W1-Humira might have the potential to bind TNF proteins at pH 7.4 but not at pH 6.0. Therefore, GaMD/CpHMD simulations generated substantially refined antibody conformations and provided possible conformation state information for predicting the binding characters at various pH values. CpHMD simulations were applied in the W1-Humira and wild-type Humira at pH 6.0 and 7.4. The CpHMD simulation results were similar to the GaMD/CpHMD simulation results. Hydrogen bonds play a unique and functionally important role in antibody/antigen interactions [53,54,55]. Comparison of W1-Humira binding modes to the wild type Humira showed that there were no hydrogen bonds in W1-Humira/TNF alpha complex stricture at pH 6.0. Our binding modes analysis also showed that W1-Humira could form hydrogen bonds with TNF alpha at pH 7.4. In vitro binding testing was applied in the W1-Humira and wild-type Humira at pH 6.0 and 7.4. Our results indicated that W1-Humira is a pH-dependent antibody, whereas wild-type Humira is not (Figure 6). Moreover, OD405 absorbance at pH 6.0 was extremely close to zero, indicating that the binding TNF alpha function of W1-Humira was turned off. The W1-Humira was 0.5-fold weaker than wild-type Humira at pH 7.4 in vitro. In vitro binding testing results were close to our GaMD/CpHMD simulations and binding modes analysis results. Therefore, we contend that our predictions yield valuable insight into effectively making predictions of antibody binding characteristics at various pH values.

## 5. Conclusions

We used cMD simulations, GaMD simulations, CpHMD simulations, and 2D free energy profiles (2D PMFs) to gain insight into the pH-dependent effects of the modified Humira (W1) at the atomic level. The 2D PMF profiles revealed the possible antibody structures at pH 6.0 and 7.4. Our predicted antibody structures seemed reasonable because our GaMD/CpHMD and binding modes analysis simulations were close to the results of in vitro testing. The W1-Humira was 0.5-fold weaker than wild-type Humira at neutral pH in vitro, and our prediction results were close to the in vitro results. Our findings increase the accuracy of antibody pH-dependent binding characteristics prediction, which may facilitate antibody drug design. Advancements in computational methods and computing power may further aid in addressing the challenges in antibody drug design.

## Figures and Tables

**Table 1:** Possible antibody conformation states based on Gaussian accelerated molecular dynamics/constant pH molecular dynamics (GaMD/CpHMD) simulations of W1-Humira at pH 6.0.

Number	Backbone Reference RMSD (BRRMSD) (Å)	Distances (D3) between the Centers of the HCDR3 and LCDR3 (Å)	PMF (Kcal/mol)
1	2.5	11.0	9.2
2	2.5	12.0	7.6
3	2.5	13.0	7.6
3	2.5	14.0	13.9
4	2.5	15.0	20.9
5	2.5	16.0	19.1
6	3.0	10.0	3.1
7	3.0	11.0	5.3
8	3.0	12.0	7.6
9	3.0	13.0	6.8
10	3.0	14.0	10.1
11	3.0	15.0	37.5
12	3.0	16.0	22.0
13	3.0	17.0	22.8
14	3.5	10.0	0.0
15	3.5	11.0	3.9
16	3.5	12.0	6.1
17	3.5	13.0	5.6
18	3.5	14.0	7.1
19	3.5	17.0	19.1
20	3.5	18.0	26.4

**Table 2:** Possible antibody conformation states based on GaMD/CpHMD simulations of W1-Humira at pH 7.4.

Number	Backbone Reference RMSD (BRRMSD) (Å)	Distances (D3) between the Centers of the HCDR3 and LCDR3 (Å)	PMF (Kcal/mol)
1	1.0	11.0	32.0
2	1.0	12.0	19.1
3	1.5	11.0	7.6
4	1.5	12.0	14.2
5	1.5	13.0	8.9
6	2.5	14.0	0.0
7	3.0	13.0	31.7
8	3.0	15.0	49.8
9	3.0	16.0	28.4
10	3.0	17.0	28.5
11	3.5	16.0	15.8
12	3.5	17.0	18.5
13	3.5	18.0	21.8

**Table 3:** Possible binding modes (residues) of W1-Humira at pH 7.4 and 6.0 with TNF alpha. (HCDR1: 30–35th residue; HCDR2: 50–66th residue; HCDR3: 99–110th residue; LCDR1: 24–34th residue; LCDR2: 50–56th residue; LCDR3: 89–97th residue).

Antibody/Regions	W1-Humira at pH 7.4	W1-Humira at pH 6.0	Humira (PDB ID: 3WD5)
HCDR1	Null	Null	Null
HCDR2	Thr52, Gly56, His57	Thr52 and His57	Thr52 and His57 (HB)
HCDR3	Leu102, Ser103 (HB), The104, Ala105 (HB)	Ser103 and Ala105	Tyr101, Ser103 and Ala105
LCDR1	Gln26, Asn30 (HB) and Tyr31	Null	Gln27, Arg30, Asn31 (HB) and Tyr32
LCDR2	Thr52	Thr52	Ala50 and Thr53 (HB)
LCDR3	His90 (HB), Asn91, Arg92 (HB) and Tyr95	Arg89, Asn91, Arg92, and Ala93	Tyr91 (HB), Asn92 (HB), and Arg93 (HB)
Other regions (Heavy chain)	Null	Null	Null
Other regions (Light chain)	Ile1, Tyr48, and Ala49	Null	Asp1

HCDR1: 30–35th residue; HCDR2: 50–66th residue; HCDR3: 99–110th residue; LCDR1: 24–34th residue; LCDR2: 50–56th residue; LCDR3: 89–97th residue. (HB: hydrogen bond).

## Data Availability

Not applicable.
